# Characterization of viroplasm formation during the early stages of rotavirus infection

**DOI:** 10.1186/1743-422X-7-350

**Published:** 2010-11-29

**Authors:** José J Carreño-Torres, Michelle Gutiérrez, Carlos F Arias, Susana López, Pavel Isa

**Affiliations:** 1 Departamento de Genética del Desarrollo y Fisiología Molecular, Instituto de Biotecnología, Universidad Nacional Autónoma de México

## Abstract

**Background:**

During rotavirus replication cycle, electron-dense cytoplasmic inclusions named viroplasms are formed, and two non-structural proteins, NSP2 and NSP5, have been shown to localize in these membrane-free structures. In these inclusions, replication of dsRNA and packaging of pre-virion particles occur. Despite the importance of viroplasms in the replication cycle of rotavirus, the information regarding their formation, and the possible sites of their nucleation during the early stages of infection is scarce. Here, we analyzed the formation of viroplasms after infection of MA104 cells with the rotavirus strain RRV, using different multiplicities of infection (MOI), and different times post-infection. The possibility that viroplasms formation is nucleated by the entering viral particles was investigated using fluorescently labeled purified rotavirus particles.

**Results:**

The immunofluorescent detection of viroplasms, using antibodies specific to NSP2 showed that both the number and size of viroplasms increased during infection, and depend on the MOI used. Small-size viroplasms predominated independently of the MOI or time post-infection, although at MOI's of 2.5 and 10 the proportion of larger viroplasms increased. Purified RRV particles were successfully labeled with the Cy5 mono reactive dye, without decrease in virus infectivity, and the labeled viruses were clearly observed by confocal microscope. PAGE gel analysis showed that most viral proteins were labeled; including the intermediate capsid protein VP6. Only 2 out of 117 Cy5-labeled virus particles colocalized with newly formed viroplasms at 4 hours post-infection.

**Conclusions:**

The results presented in this work suggest that during rotavirus infection the number and size of viroplasm increases in an MOI-dependent manner. The Cy5 in vitro labeled virus particles were not found to colocalize with newly formed viroplasms, suggesting that they are not involved in viroplasm nucleation.

## Background

Rotaviruses are the major cause of severe diarrhea in children and young animals worldwide. As a members of the family *Reoviridae*, they have a genome of 11 segments of double-stranded RNA (dsRNA) enclosed in three protein layers, forming infectious triple-layered particles (TLP) [1]. During, or just after entering the cell's cytoplasm, the outer capsid, composed of VP4 and VP7, is released, yielding transcriptionally active double-layered particles (DLP). The produced viral transcripts direct the synthesis of viral proteins and serve as templates for the synthesis of negative-RNA strands to form the genomic dsRNA. During the replication cycle of rotavirus electron-dense cytoplasmic inclusions, named viroplasms, are formed [2]. Such cytoplasmic inclusions are observed during infection with a number of animal viruses [3], including reoviruses, as other members of the *Reoviridae *family [4].

In rotaviruses two non-structural proteins, NSP2 and NSP5, have been shown to be sufficient to form membrane-free cytoplasmic inclusions, which are known as viroplasms-like structures [5]. *In vivo *immunofluorescence visualization of viroplasms shows they are heterogeneous in size [6,7]. It is in these structures where the synthesis of dsRNA and its packaging into pre-virion core particles take place [8]. Besides NSP2 and NSP5, other viral proteins accumulate in viroplasms - namely VP1, VP2, VP3, VP6, and NSP6 [7,9-11]. The key role of NSP2 and NSP5 proteins in the formation of viroplasms has been demonstrated by knocking-down their expression by RNA interference, which results in the inhibition of viroplasm formation, genome replication, virion assembly, and a general decrease of viral protein synthesis [7,8,12]. Viroplasm formation has been studied using electron or fluorescence microscopy [6,13-15], however, despite their importance in the replication cycle of rotavirus, little is know about their dynamics of formation. The observation that bromouridine-labeled RNA localizes to viroplasms suggested that the viral transcripts are synthesized within viroplasms, which led to the hypothesis that the entering viral particles could serve as points of nucleation for the formation of viroplasms [8]. In this work, the dynamics of viroplasm formation in MA104 cells infected with rotavirus strain RRV was studied as a function of time and multiplicity of infection (MOI). Using fluorescently labeled purified rotavirus particles; we showed that the incoming TLPs do not seem to be involved in the formation of viroplasms.

## Materials and methods

### Cells, viruses, antibodies, and fluorophores

MA104 cells were cultured in Advanced Dulbecco's Modified Eagle's Medium (DMEM) supplemented with 3% fetal calf serum (FBS). The rhesus rotavirus strain RRV, obtained from H.B. Greenberg (Stanford University, Stanford CA), was propagated in MA104 cells. The rabbit polyclonal serum to NSP2 protein has been described previously [16]. Horseradish peroxidase-conjugated goat anti-rabbit polyclonal antibody was from Perkin Elmer Life Sciences (Boston, MA), Alexa 488 and 568 -conjugated goat anti-rabbit polyclonal antibodies, FluoSpheres carboxylate-modified microspheres, 0.1 μm, yellow-green fluorescent (505/515), were from Molecular Probes (Eugene, OR), and Cy™5 Mono-Reactive Dye pack was from Amersham, GE Healthcare, UK.

### Identification, quantitation and size analysis of viroplasms

MA104 cells grown in 10 mm coverslips were infected with rotavirus strain RRV at different MOI's for 1 hour at 4°C. After washing unbound virus, the cells were incubated at 37°C for different times post-infection. The cells were fixed with 2% paraformaldehyde, and permeabilized with 0.5% Triton X-100 in PBS containing 1% bovine serum albumin, as described previously [17]. Cells were then incubated with rabbit polyclonal sera to NSP2 protein, followed by staining with goat anti-rabbit IgG coupled to Alexa-488 or 568. The images were acquired using a Zeiss Axioskop 2 Mot Plus microscope and analyzed by Image Pro Plus 5.0.2.9 and Adobe Photoshop 7.0. All images were acquired with a 60× objective, with a real time CCD Camera in 256 grey scales, and the size of the images was 1392 × 1040 pixels, with 8 bits. The estimation of viroplasm size was done using the Analyze particle function of Image J 1.32j program (Wayne Rasband, NIH, USA).

### Immunodetection of rotavirus NSP2 protein

MA104 cells grown in 24-well plates were infected with rotavirus strain RRV at different MOI's for 1 hour at 4^o^C. After washing unbound virus, the cells were incubated at 37^o^C for different times post-infection. At the indicated time points, the cells were washed twice with PBS and lysed with Laemmli sample buffer. Proteins were separated by 10% SDS-PAGE and transferred to nitrocellulose membranes (Millipore, Bedford, MA). Membranes were blocked with 5% non-fat dried milk in PBS, and incubated at 4^o^C with primary anti NSP2 polyclonal antibody in PBS with 0.1% milk, followed by incubation with secondary, horseradish peroxidase-conjugated antibodies. The peroxidase activity was revealed using the Western Lightning™Chemiluminiscence Reagent Plus (Pelkin Elmer Life Sciences). The images obtained were scanned and the band densities analyzed using Image pro software.

### Conjugation of virus with fluorophore and colocalization of labeled viruses with viroplasms

To label virus with fluorophores, RRV virions were purified by cesium chloride gradient centrifugation as described previously [18]. The purified TLP's of simian strain RRV were washed twice with 10 mM Hepes pH 8.2, 5 mM CaCl_2_, 140 mM NaCl, and labeled with Cy5 mono reactive dye (0.1, 0.5, 1, 2.5, and 5 nmol of fluorophore for 1 μg of purified virus) at room temperature for 1 hour with gentle agitation. The reaction was stopped by addition of Tris-HCl pH 8.8 to a final concentration of 50 mM. Labeled viruses were separated from unbound fluorophore by gel filtration on a G25 sepharose column. As control, the purified TLP's of strain RRV were processed in identical way without addition of fluorophore. Viral titres were determined by a standard immunoperoxydase assay as described previously [19]. DLP's were prepared by EDTA treatment of labeled TLP's. To determine which viral proteins were conjugated with fluorophore, labeled and non-labeled TLP's and DLP's were resolved in PAGE gel, analyzed on Typhoon-Trio (Amersham Biosciences) and stained by silver nitrate. Labeled particles were compared with FluoSpheres [carboxylate-modified microspheres, 0.1 μm, yellow-green fluorescent (505/515)] using confocal microscope LSM-510 Zeiss, mounted on inverted microscope Zeiss Axiovert 200 M, with AIM software, using objective Plan-neofluor 100×/1.30 Oil Ph3 (Carl Zeiss). To detect green staining, excitation laser Argon 2 488 nm was used with emission filter BP 500-530 nm, and for far red staining laser Helio-Neon 633 nm was used with emission filter BP650-670 nm. To colocalize labeled TLP's with viroplasms, MA104 cells grown on coverslips were infected with Cy5 -labeled RRV TLP's (MOI of 2) for 1 hour at 37°C. After washing unbound virus, the infection was left to proceed for 4 hours, and then the cells were fixed and the viroplasms were detected as described above using a rabbit polyclonal antibody specific for rotavirus NSP2 protein, and a goat anti rabbit IgG coupled to Alexa 488. Images were acquired using a confocal microscope as described above, as stacks of 10 images 800 nm thick, with resolution of 1024 × 1024 pixels, and processed by nearest neighbor deconvolution using AIM software. Acquired images were processed by Image J 1.32j and Adobe Photoshop 7.0. Analyzing corresponding individual images ensured localization of all Cy-5 labeled viral particles inside cytoplasm.

## Results

### The number of viroplasms and the level of the NSP2 protein increase during rotavirus infection, in direct correlation with the multiplicity of infection

**Figure 1:**
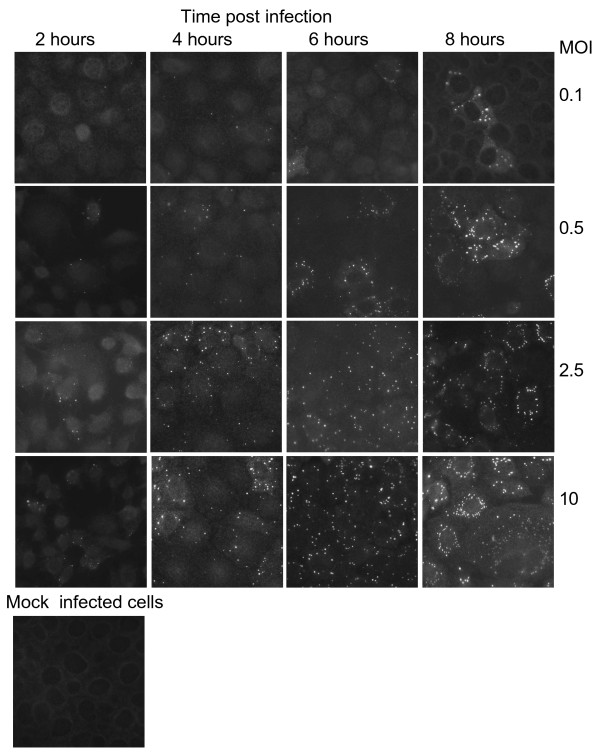
**Detection of viroplasms in cells infected at different MOI's, and at distinct times post infection**. MA104 cells were infected with RRV at the indicated MOI, and at different times post infection at 37°C, the cells were fixed and immunostained with a rabbit antibody to NSP2 and a goat anti-rabbit antibody coupled to Alexa-488 or Alexa-568. Images were acquired using Zeiss Axioskop 2 Mot Plus microscope and Image Pro Plus 5.0.2.9 program. Mock-infected cells are shown as control.

**Figure 2:**
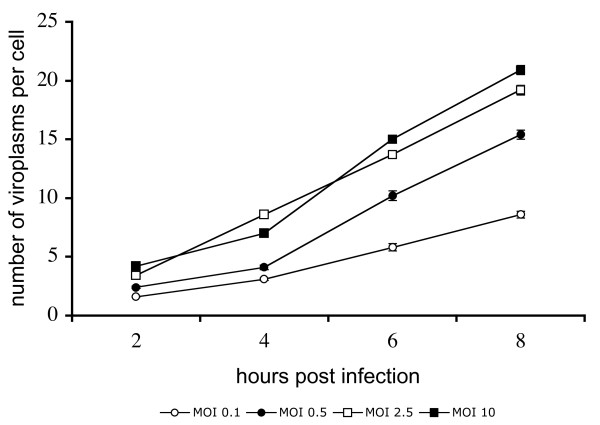
**The number of viroplasms per cell increases with time of infection**. MA104 cells were infected at different MOI's as described in Figure 1, and viroplasms were detected by immunofluorescent staining of NSP2. Viroplasms were counted in 400 infected cells in each condition. Each value is expressed as mean ± standard error. The increase in the number of viroplasms during the infection at each MOI, and the differences in the number of viroplasms between different MOI at each time point were statistically significant (P < 0.05, student T-test).

It has been described that viroplasms can be visualized in rotavirus infected cells by immunofluorescence as early as 2 hours postinfection (hpi) [14]. Therefore, to learn about the kinetics of viroplasm formation at early stages of rotavirus infection, MA104 cells grown in coverslips were infected with rotavirus strain RRV at different MOI's, and at different times post-infection (2, 4, 6, or 8 hours) the cells were fixed and the viroplasms were detected by immunofluorescence using a mono-specific serum to NSP2. Viroplasms were detected as early as 2 hpi as discrete dots that were not observed in control, mock infected cells, and their number and size increased as the infection proceeded (Figure 1). To quantitate the increase in the number of viroplasms during infection, the number of viroplasms in 400 infected cells from each condition was scored. It was observed that independently of the MOI used, the number of viroplasms per cell increased as the infection proceeded (P < 0.05, Student's t-test), almost duplicating every two hours up to 6 hours (Figure 2).

**Figure 3:**
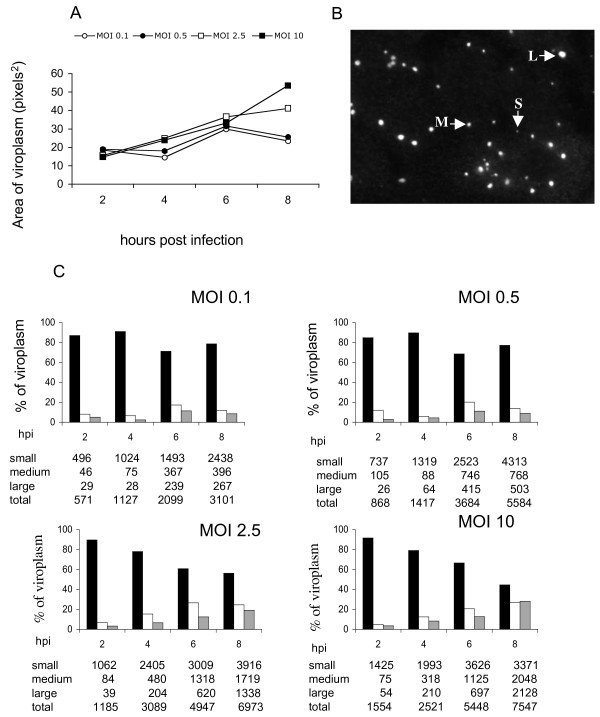
**The proportion of larger viroplasms increases during rotavirus infection**. (A) MA104 cells were infected at different MOI's as described in Figure 1, and the area of each viroplasm was estimated by pixel determination using Image J. The same images used in Figure 2 were analyzed for this figure. Each value represents the mean ± standard error of viroplasms detected in 360 cells, in pixels^2. ^(B) Based on a microscopic comparison, viroplasms were divided into three arbitrary groups, small (S) (4-33 pixels^2^), medium (M) (34-69 pixels^2^), and large (L) (70-200 pixels^2^). Arrows point to viroplasms representative of each size S, M, and L. (C) Relative amounts of small, medium and large viroplasms during the course of infection at different MOI's. Bars represent the proportion of viroplasms for each multiplicity of infection, (black bars - small; white bars medium; grey bars large viroplasms) with 100% being the total number of viroplasms counted in 360 cells. The numbers under each graph represent the number of the different classes of viroplasms found in each condition analyzed.

To determine the size of viroplasms, their area was determined in pixels^2 ^in 360 infected cells per condition. While at higher MOI's (2.5 and 10) there was a constant increase in the average size of the viroplasms, at low MOI's (0.1 and 0.5) a fluctuation in the viroplasm size was observed (Figure 3A). To analyze the size of the viroplasms in more detail, viroplasms were divided into three arbitrary groups: small (4 - 33 pixels^2 ^), medium (34 - 69 pixels^2^), and large (70 - 200 pixels^2^) (Figure 3B). Throughout the course of infection, and independently of the MOI used, or the time post infection analyzed, the population of small viroplasms predominated in number and also in proportion of all viroplasms (Figure 3C). Differences were observed when the size of viroplasms was compared in cells infected with low (0.1 and 0.5) or high (2.5 or 10) MOI's. While at high MOI's there was a gradual decrease in the proportion of small viroplasms during the course of infection, from 90 and 92% (2 hpi) to 56 and 45% (8 hpi) respectively, with a concomitant increase in the medium and large size viroplasms, the proportion of small viroplasms at low MOI's was more stable (Figure 3C). This suggests that while at high MOI's small viroplasms might convert to larger size viroplasms, probably due to large amount of protein synthesized in cell, at low MOI's the formation of new small viroplasms prevails, and they could become larger at later times post infection, however, this possibility was not investigated in this work.

**Figure 4:**
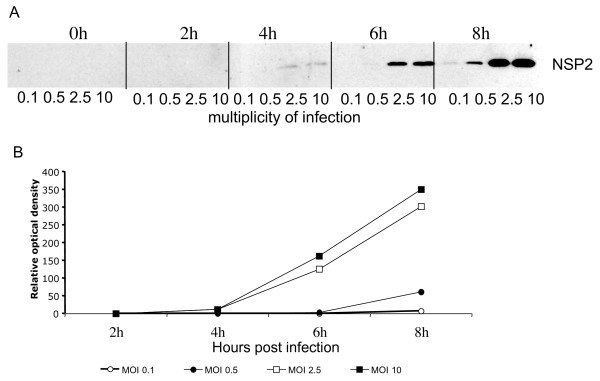
**The amount of NSP2 protein increases with time of infection**. MA104 cells were infected with RRV at the indicated MOI, and at different times post-infection at 37°C, the cells were harvested in Laemmli buffer. Equal amount of cell lysates were separated by SDS-PAGE and blotted onto nitrocellulose. (A) The expression of rotavirus NSP2 protein was determined by immunostaining with a rabbit antibody to NSP2 and a goat anti-rabbit antibody coupled to peroxydase. A representative experiment from three carried out is shown. (B) Optical density of the protein bands shown in A, as determined by scanning and analysis using Image pro.

To determine if the number and size of viroplasms correlate with the level of NSP2 synthesized during infection, cells infected at different MOI's were harvested at different times post-infection, and the level of NSP2 was determined by Western blot (Figure 4). While at high MOI's (2.5 and 10) the NSP2 protein was detected at 4 hours post-infection, and increased as infection proceeded, at low MOI's (0.1 and 0.5) the NSP2 protein was detected until 8 hours post-infection (Figure 4A). Densitometric analyses showed that the dynamics of accumulation of NSP2 during infection with high MOI's (Figure, 4B) was similar to that observed for the increase in the number of viroplasms (Figure 2).

### Viroplasms do not colocalize with fluorescently labeled particles

**Figure 5:**
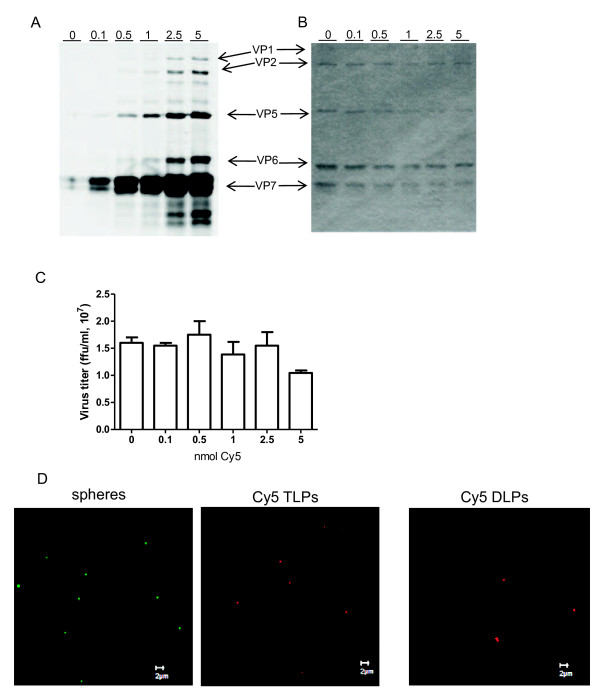
**Conjugation of viral particles with Cy5 monoreactive dye**. Purified TLP's of strain RRV were conjugated with different amounts of Cy5 monoreactive dye as described under Materials and methods. The reaction was stopped by Tris-HCl and labeled viruses were separated by gel filtration on G25 sepharose column. (A) Labeled and non-labeled viral particles were separated on 10% PAGE, and gel was visualized on Typhoon Trio to determine Cy5 - viral protein conjugation. (B) Same gel as shown in A was stained using silver nitrate. Viral proteins are identified by arrows. (C) MA104 cells grown in 96 well plates were infected with labeled and non labeled viral preparations, and 14 hours post infections cells were fixed and infected cells were detected using peroxydase immuno staining with anti-rotavirus polyclonal antibodies. Results are expressed as number of viral infectious focus forming units per ml. (D) Comparison of fluorophore labelled TLP's and DLP's (prepared by EDTA treatment), shown in red, with 100 nm Fluorosferes, shown in green, observed in confocal microscope.

**Figure 6:**
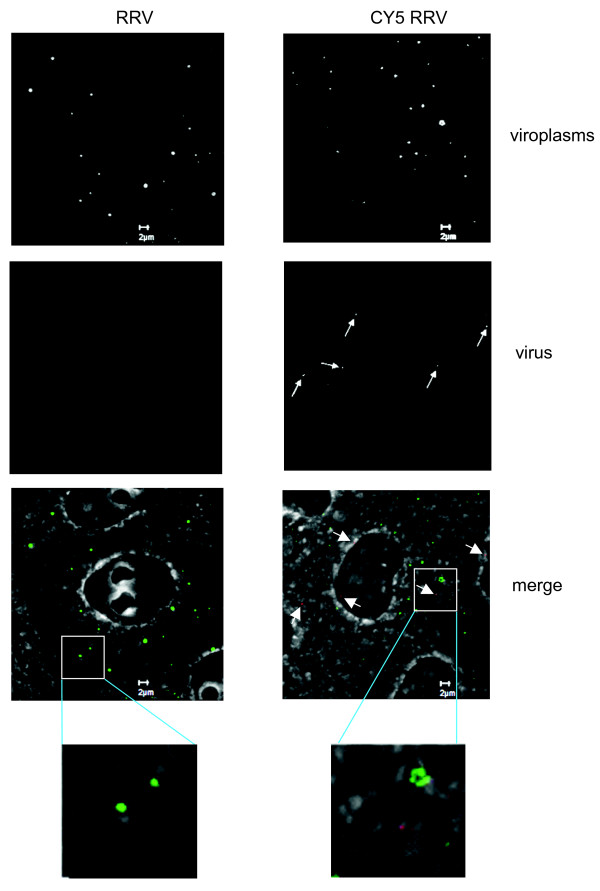
**Viroplasms do not colocalize with Cy5 labeled infectious rotavirus particles**. MA104 cells grown in coverslips were infected with purified rotavirus strain RRV TLPs (left side panels) or purified rotavirus strain RRV TLP's labeled with Cy5 (right side panels). Four hours after infection, cells were fixed, and the viroplasms detected with an anti-NSP2 polyclonal antibody, and a secondary antibody coupled to Alexa-488. Images were obtained with confocal microscope LSM 510 and processed as described in material and methods. In merge, detected viroplasms are in green, and Cy5 labeled RRV particles are in red, pointed by arrows. Detail of viroplasm location and Cy5 labeled RRV are shown.

It has been previously described that virus-like particles produced in insect cells by the co-expression of rotavirus capsid proteins VP6, VP4, VP7, and a VP2 protein fused to GFP or to DsRed protein, can be visualized in living cells [20]. Other viruses have also been observed in cells after being directly labeled with fluorophores; among these are influenza A virus, poliovirus, dengue virus, and SV40 [21-24]. To determine if the formation of viroplasms is nucleated by the entering viral particles, purified infectious TLP's of RRV were conjugated with Cy5. Analysis of viral proteins by PAGE showed that all proteins were conjugated. Importantly the intermediate capsid protein VP6 was efficiently labeled, ensuring that viral particles will be visible even after loss of the outer capsid proteins VP7 and VP4 (or its trypsin cleavage products VP5 and VP8) (Figure 5A and 5B). Viral titre after conjugation was similar to mock conjugated virus, observing a small decrease of infectivity when using 5 nmol of Cy5 for conjugation, suggesting that viral infectivity was not compromised (Figure 5C), therefore, for the following experiments 1 nmol of Cy5/μg of virus was chosen. Most importantly, both TLP's and DLP's (prepared from TLP's by EDTA treatment), labeled with 1 nmol of Cy5, were comparable to 100 nm Fluorospheres when observed in confocal microscopy (Figure 5D). Since it was possible to visualize the fluorescently labeled viral particles, we used them to observe their intracellular distribution with respect to the newly formed viroplasms. To do this, MA104 cells grown in coverslips were infected with Cy5-conjugated RRV TLP's at an MOI of 2, and 4 hpi the cells were fixed, the viroplasms were immunostained using a polyclonal sera to NSP2, and images were acquired using confocal microscopy, as described under material and methods. Fluorescently labeled viral particles were observed distributed in the cytoplasm as discrete spots (Figure 6). The number of labeled viral particles, viroplasms, and their co-localization was counted independently by two persons in 31 cells. In these, 117 labeled virus particles and 467 viroplasms were observed, however, only 2 of the viral particles observed colocalized with viroplasms, while the rest appeared independent of each other in the cell cytoplasm.

## Discussion

The formation of viroplasms has been previously studied using electron and fluorescence microscopy, however, those studies have focused only on late (4 to 24 hpi) stages of infection [6,13,15]. Only Eichwald et al [14] have studied earlier stages of viroplasm formation, and in their work, following the expression of an NSP2 protein fused to EGFP in rotavirus SA-11 infected cells, they observed that the total number of viroplasms decreased with time, with a concomitant increase in their size, starting at 6 hpi. This observation was interpreted as fusion events between smaller viroplasms. Similar results were reported by Cabral-Romero and Padilla-Noriega [15] using the strain SA-11 in BSC1 cells, although at even later (10 hpi) stages of infection. Comparing the formation of viroplasms between SA-11 and OSU rotavirus strains, Campagna et al. [6] observed that the viroplasms formed in OSU infected cells did not increase in size as readily as those formed during infection with SA-11. In this work, after infection with rotavirus strain RRV, using different MOI's, an increase in the number of viroplasms and in the amount of the NSP2 protein was observed. The size of viroplasms was observed to increase when higher MOI's were used.

There are several possibilities to explain the discrepancies reported. First, the decrease in the number of viroplasms was observed only during infection with strain SA-11 [14,15], but not with strains OSU [12], and RRV (this work). It is known that some viral functions (receptor specificity, plaque formation, extraintestinal spread, IRF3 degradation, etc) may vary among different rotavirus strains [25-28] what opens the possibility that there could also be strain-specific differences for viroplasm formation. In fact, an impaired phosphorylation of NSP5 affected differently the morphogenesis of viroplasms in cells infected with either SA-11 or OSU rotavirus strains [6]. The differences observed between our studies and those of other groups could also arise from the different methodologies used to detect viroplasms. While in our case the newly synthesized rotavirus proteins were immunodetected and analyzed in 400 cells, in the study by Eichwald et al. [14] the identification of viroplasms was based on the detection of NSP2-EGFP or NSP5-EGFP fusion proteins in 20 cells. It is possible that the large amount of recombinant fusion proteins that accumulated in the cytoplasm of transfected cells before rotavirus infection could change the kinetics of viroplasm formation, since upon rotavirus infection a rapid redistribution of the EGFP - proteins was observed. It was not possible to compare the exact number of viroplasms obtained in that study, since the MOI that was used to infect the transfected MA104 cells was not mentioned.

In this work, studying the kinetics of viroplasm formation during the infection of strain RRV, we observed an increase in both the number and size of viroplasms with time and this increment was dependent on the MOI used. At high MOI's (2.5 and 10) the increase correlated with the amount of NSP2 protein detected at a given time point, while at lower MOI's (0.1 and 0.5), the smaller increase in NSP2 protein correlated with a less variable viroplasm size. It is possible that when a critical concentration of NSP2 and NSP5 is reached, and as other viral proteins accumulate, viroplasms start to form, first as small entities, and then becoming larger at later stages of the replication cycle. Although, it is not possible to determine if the increase in size is caused by fusion of smaller viroplasms or by addition of newly produced rotavirus proteins to small viroplasms, our observations are more consistent with the idea that new small viroplasms are generated constantly during the replication cycle, since even at later stages of infection a large proportion of small viroplasms was observed. It remains to be determined if the small viroplasms, presumably generated by the aggregation of NSP2 and NSP5 require an additional priming signal, or if it is only the concentration of free NSP2 and NSP5 what dictates the formation of a new viroplasms.

The mechanism of viroplasms formation and its protein content is unknown. The fact that viroplasms are sites for rotavirus transcription at late stages of infection (8.5 hpi) led to the so far unproven hypothesis that incoming DLP's serve as focal points of viroplasm assembly [8]. In this work we tested this hypothesis by visualization of incoming viral particles and by analyzing their colocalization with newly formed viroplasms. Only 2 out of 117 CY5-conjugated viral particles observed in 31 cells colocalized with viroplasms, suggesting that the entering virus particles do not serve as focal points for accumulation of the newly synthesized proteins into viroplasms.

In addition, if the entering virus particles served as focal point for viroplasm formation, the number of viroplasms at early times of infection should correspond to the estimated number of infectious viral particles that entered the cell. However, a correlation between the number of viroplasms detected at early times post-infection and the expected number of infectious particles entering the cells, according to the Poisson distribution (Table 1), was not observed (Figure 2). At low MOI's, when 95% and 77% of infected cells are expected to be infected with only 1 viral particle (with MOI's of 0.1 and 0.5 respectively), there were more viroplasms per cell [1.6 and 3.1 for a MOI of 0.1 and 2.4 and 4.1 for an MOI of 0.5 (2 and 4 hpi respectively)], while at an MOI of 10, when 87% of the cells are expected to be infected with 7 or more infectious viral particles, only 4.2 and 7 viroplasms were observed at 2 and 4 hours post-infection (Figure 2). These results suggest that at the onset of infection the entering viral particles do not serve as nucleation centers for the formation of viroplasms as suggested [8]. The fact that the plasmid expression of NSP2 and NSP5 proteins alone, in absence of infectious virus, are able to form viroplasm-like structures also supports this conclusion.

**Table 1:** Theoretical percentage of cells infected with a given number of viral particles at different multiplicities of infection, as determined by the Poisson distribution, with 100% being all infected cells.

	Multiplicity of infection			
No. of infectious viral particles/cell	0.1	0.5	2.5	10
1	95.1*	77.1	22.4	0.05
2	4.7	19.3	27.9	0.2
3	0.2	3.2	23.3	0.8
4	0.003	0.4	14.6	1.9
5	0†	0.04	7.3	3.8
6	0†	0.003	3.0	6.3
7	0†	0.0002	1.1	9.0
8	0†	0†	0.3	11.3
9	0†	0†	0.09	12.5
10	0†	0†	0.02	12.5
11	0†	0†	0.005	11.4
12	0†	0†	0.001	9.5
13	0†	0†	0.0002	7.3
14	0†	0†	0†	5.2
≥15	0†	0†	0†	8.3
% of total cells infected	9.5	39.3	91.8	99.8

* % of infected cells

† < 0.0001%

Recently it was suggested that rotavirus viroplasms could interact with microtubules [15]. NSP2 was also shown to interact with tubulin, inducing the collapse of the microtubule network, and viroplasms were shown to colocalize with tubulin granules [29]. Similar interaction of reovirus viral inclusion bodies with microtubules [30] suggests the possibility that tubulin could have a more general role in the replication cycle of viruses of the *Reoviridae *family.

Although viroplasms play a crucial role in rotavirus replication and assembly, the factors that govern their formation and function, are still not clearly understood. The development of live cell imaging tools should provide more detailed information about these processes.

## 4. Conclusions

Rotavirus replication takes place in electrodense structures known as viroplasms, however, little is known about their dynamics of formation, and the factors that drives viroplasm nucleation. The results presented in this work show that during rotavirus infection the number and size of viroplasms increases steadily with time, and depends on the MOI used. Using in vitro Cy5 - labeled infectious viral particles we observed that the entering viruses do not seem to be involved in viroplasm nucleation. It is possible that some cellular protein, like tubulin, are required for this process, however much work is needed to characterize in detail this essential step of rotavirus replication.

## List of abbreviations

DLP: double layered particles; DMEM: dulbecco's modified eagle medium; DSRED: Discosoma sp. Red fluorescence protein; DSRNA: double stranded RNA; EGFP: enhanced green fluorescence protein; GFP: green fluorescence protein; HPI: hours post infection; IRF3: interferon regulatory factor 3; MOI: multiplicity of infection; NSP2: nonstructural protein 2; NSP5: nonstructural protein 5; NSP6: nonstructural viral protein 6; PAGE: polyacrylamide gel electrophoresis; TLP: triple layered particles; VP1: structural viral protein 1; VP2: structural viral protein 2; VP3: structural viral protein 3; VP4: structural viral protein 4; VP5: structural viral protein 5; VP6: structural viral protein 6; VP7: structural viral protein 7; VP8: structural viral protein 8.

## Competing interests

The authors declare that they have no competing interests.

## Authors' contributions

JJCT carried out study of kinetics of viroplasms formation, started analysis of fluorophore conjugated viral particles, MG carried out Cy5-TLP's: viroplasm colocalization studies, CFA: has been involved in data analysis and revising final manuscript, SL participated in designing of the study and in critical reading of manuscript, PI conceived of the study, has been involved in Cy5-TLP's: viroplasms colocalization, interpretation of results and drafted the manuscript. All authors read and approved the final manuscript.
